# Assessment of diagnostic reference levels for paediatric cardiac computed tomography in accordance with European guidelines

**DOI:** 10.1007/s00411-023-01031-6

**Published:** 2023-06-22

**Authors:** Mohamed S. Aboul Hamad, Ehab M. Attalla, Hanan H. Amer, Mohamed M. Fathy

**Affiliations:** 1 Biophysics Department, Faculty of Science, Cairo University, Giza, Egypt; 2 Radiology Department, ALNas Hospital, Cairo, Egypt; 3 National Cancer Institute, Cairo University, Giza, Egypt

**Keywords:** Diagnostic reference levels, Cardiac computed tomography, Paediatric, Effective dose, Patient exposure

## Abstract

Recently, paediatric cardiac computed tomography (CCT) has caused concerns that diagnostic image quality and dose reduction may require further improvement. Consequently, this study aimed to establish institutional (local) diagnostic reference levels (LDRLs) for CCT for paediatric patients, and assess the impact of tube voltage on proposed DRLs in terms of the volume computed tomography index (CTDIvol) and dose length product (DLP). In addition, effective doses (EDs) of exposure were estimated. A population of 453 infants, whose mass and age were less than 12 kg and 2 years, respectively, were considered from January 2018 to August 2021. Based on previous studies, this number of patients was considered to be sufficient for establishing LDRLs. A group of 245 patients underwent CCT examinations at 70 kVp tube voltage with an average scan range of 23.4 cm. Another set of 208 patients underwent CCT examinations at 100 kVp tube voltage with an average scan range of 15.8 cm. The observed CTDI_vol_ and DLP values were 2.8 mGy and 54.8 mGy.cm, respectively. The mean effective dose (ED) was 1.2 mSv. It is concluded that provisional establishment and use of DRLs for cardiac computed tomography in children are crucial, and further research is needed to develop regional and international DRLs.

## Introduction

Congenital heart disease (CHD) is considered to be one of the most common birth defects and the major cause of death in infants under the age of 1 year (Lee et al. 2012; Bouma et al. 2017; Van Der Linde et al. 2011). Although invasive catheter-directed cardiac angiography is now the gold standard for diagnostic assessment of complicated cardio-vascular anomalies in infants, extended fluoroscopic and cine evaluations have still the potential to expose patients to high doses of radiation and contrast medium. Additional drawbacks of these modalities include patient discomfort, a lack of availability, and high costs. The improved spatial and temporal resolution of multi-detector computed tomography (MDCT) has enabled non-invasive examination of intrathoracic vessels, cardiac architecture, and coronary arteries in newborns with congenital heart disease (Lee et al. 2012; Tsai et al. 2008). As a consequence, cardiac computed tomography angiography (CTA) in infants has evolved to play a crucial role in assessing complex CHD.

In the past 15 years, the use of computed tomography (CT) and interventional radiology (IR) procedures has increased drastically (Granata et al. 2019). For this reason, due to the increased risk of cancer induction radiation exposure from CTA has become an essential consideration.

Recently, children's radiological imaging, particularly of radiation-sensitive organs, has been classified as among the research fields with the greatest rate of growth (Pearce et al. 2012). Children are more vulnerable (UNSCEAR 2013) to the harmful effects of radiation than adults, making paediatric exams a particular source of concern. Recent studies have shown an increase in cancer incidence following paediatric CT scans (Tajaldeen et al. 2022). Due to a longer life expectancy and higher radiation sensitivity, children are suffering a higher risk of radiation damage than adults (Tsai et al. 2008). Therefore, particular attention should be paid on the justification and optimisation of paediatric CT examinations via establishing and using diagnostic reference levels (DRLs) in paediatric radiology (Bosmans et al. 2018).

In 1999, the "Guidance on diagnostic reference levels (DRLs) for medical exposure" was published by the European Commission (European Commission 1999). This study emphasises the necessity of developing DRLs for high-dose medical examinations of radiation-sensitive patients, particularly children, including CT and interventional radiology (IR). The International Commission on Radiological Protection (ICRP 2017) and the European Council Directive 2013/59/Euratom Basic Safety Standards (BSS) recommend and mandate the establishment and use of DRLs (European Society of Radiology 2015). DRLs are a valuable tool for optimizing patient doses in diagnostic and interventional radiology. Furthermore, the fact that CT examinations contribute a significant portion of the population dose from all diagnostic uses of ionizing radiation makes it clear that the necessity for DRLs in CT is critical (European Commission 2014; Damilakis et al. 2014).

Despite the recommendations and the clear need for DRLs for paediatric examinations, few paediatric DRL data are available that have been established within some European countries (Bosmans et al. 2018). Due to the general paucity of patient dose data for paediatric examinations as well as due to the fact that patient dose levels vary considerably with age and weight of the patients, DRLs for several age, size or weight groups need to be defined (Muhammad et al. 2020).

Taking into account and according to the ICRP recommendations (ICRP 2001, 2007) DRLs are not to be used to enforce restrictions on individual patient overdoses, and neither their regulatory nor their commercial use is intended. In contrast, DRLs allow to ensure that the doses administered to patients are keeping with the ALARA principle (as low as reasonable achievable). Examination-specific DRLs can support practises in tracking and enhancing patient protection. It can be anticipated that paediatric DRLs will increase exposure awareness and prompt paediatric practises to more actively manage the imaging quality required for children examinations. The European Commission acknowledged this need in December 2013 when it initiated the PiDRL project to build European DRLs for paediatric patients (Bosmans et al. 2018; ICRP 2001, 2007).

According to European Guidelines on DRLs for Paediatric Imaging, DRLs are categorized in three sub-types as follows: Local DRL, National DRL, and Regional DRL. “Local DRL (LDRL) is based on the 3rd quartile (the 75th percentile) value of the distribution of patient doses obtained from radiology departments in a single large healthcare facility or a group of healthcare facilities, for a defined clinical imaging task (i.e., common indication-based protocol) surveyed for standardised patient groupings” (Bosmans et al. 2018).

The ICRP recommends the use of DRLs as a benchmark for local practise in support of managing patient doses and promoting optimization. Additionally, the ICRP uses the term "typical value" to provide a local comparator to national reference levels, if available, in a similar manner to local DRLs. Typical values serve as a guide to encourage further optimization in a facility and may be set for a single piece of equipment in a hospital to provide a comparator for an emerging technology or technique. Overall, typical values are helpful in promoting further optimization for a facility (ICRP 2017).

The present work aimed to promote the establishment and use of DRLs in infants’ radio-diagnostic imaging practises to advance optimizations of radiation protection of paediatric patients. Moreover, it purposed to develop diagnostic reference ranges (DRRs) and a method for an individual practise to calculate site-specific reference doses for CT scans of cardiac imaging in infants, and to recommend a methodology for establishing and using DRLs for CT paediatric radio-diagnostic imaging (Kadir et al 2022).

## Materials and methods

### Data collection

A retrospective review of 453 paediatric patients (53.52% males and 46.48% females) with age ranges of 0–2 years and mass ranges of 0–12 kg was conducted. From January 2018 to August 2021, data were collected in the radiology department of a single tertiary care children's hospital.

### CT examinations, equipment specification, and quality assurance

The radiological departments were equipped with two CT devices (Siemens, SOMATOM, Healthineer, Germany) of different versions (one is Flash and the other is Drive version). Both devices included a dual x-ray source scanner and stellar detectors. The technical specifications of the equipment, such as temporal resolution, slice acquisition, spatial resolution, and maximum scan speed, were the same at both CT protocols, and tube voltages of 70 and 100 kVps were used.

A CT protocol is a set of parameters (i.e. mAs, kVp, rotation time, pitch, slice acquisition, scan range, etc.) that specify specific exam requirements. Cardiac CT image acquisition fundamentally differs from most routine CT exams in that, in addition to being triggered by breathing, it is also triggered by an electrocardiogram (ECG). ECG gating just means that CT data collection is timed to a certain phase of the heartbeat. The phase is measured as a percentage of the R-R interval. For example, if a person's heart rate is 60 beats per minute (bpm), 30% of the time is near the end of systole and 75% of the time is near the end of diastole.

All CCT procedures were performed using automatic tube current modulation (ATCM) to obtain an optimized image quality for varying local patient attenuations throughout a scan.

During this study, 245 paediatric CCT examinations were scanned using the dual source SOMATOM Drive CT at 70 kVp tube voltage with an average scan range of 23.4 cm, while the other 208 paediatric CCT examinations were performed with the dual source SOMATOM Flash that operated at 100 kVp tube voltage with an average scan range of 15.8 cm.

Quality control (QC) measurements were periodically performed by a medical physicist to ensure consistent and acceptable diagnostic image quality, as recommended by the manufacturer's manual. All acquired CCT images were evaluated by experienced radiologists in radiology departments, and diagnostically acceptable exams were included in this study.

Demographic patient information (weight, height, sex, and age) and scan parameters of retrospective ECG-gating exams, including type of equipment, tube voltage (kVp), average tube current, use of tube current modulation, number of detector rows, detector width of the CT scanner, rotation time (s), and helical/axial scanning, were recorded.

### Effective dose calculations

The effective patient dose (ED) is an important dose quantity associated with the probability of health damage due to stochastic effects, which takes into consideration the relative radiosensitivity of the internal tissues in the scanned region. ED is a derived quantity that can be used for comparative purposes between studies.

Effective dose, in *mSv*, was estimated by multiplying DLP with the cardiac conversion factor (k factor) recommended in (ICRP 2007) and Trattner (Trattner et al. 2018) (Eq. 1). In the present study, the conversion factor at 70 kVp was not available; consequently, the conversion factor at 80 kVp was used.

1 $$ {\text{ED}}\left( {{\text{mSv}}} \right) = {\text{DLP}}\left( {{\text{mGy}}{\text{.cm}}} \right) \times k\left( {\frac{{{\text{mSv}}}}{{{\text{mGy}}{\text{.cm}}}}} \right) $$

### Statistical analysis

According to European guidelines, it is recommended that CTDIvol and DLP are determined for a 32 cm phantom for all paediatric body CT examinations (chest, abdomen, trunk and spine) and for a 16 cm phantom for paediatric head CT examinations. Therefore, in the present study the CTDIvol and DLP values were related to a 32 cm phantom and directly collected from the CT machine.

The distribution of CTDI_vol_ and DLP values were assessed using the SPSS V.22.0 software. A *P* value < 0.05 was considered statistically significant. The minimum, median, mean, maximum, 25th percentile, 75th percentile (set as DRL) and interquartile range (IQR) were assessed to develop diagnostic reference ranges (DRRs), for each protocol in the present study based on the ICRP 103 recommendation (Protection 2007). The DRR concept was introduced by the ICRP to balance the benefits for a patient, the required diagnostic data for the medical procedure, and the patient's radiation dose. The DRR covers the 25–75% range of dose distribution, with the 75th percentile identified as the maximum limit and the 25th percentile as the lower limit. In 2017, the ICRP recommended the use of the 75th percentile as a kind of investigation level known as the DRL (Kadir et al. 2022).

The Mann–Whitney *U* test and boxplots were used to compare proposed DRLs based on estimated CTDIvol and DLP values at tube voltages of 70 and 100 kVp.

## Results

**Fig. 1:**
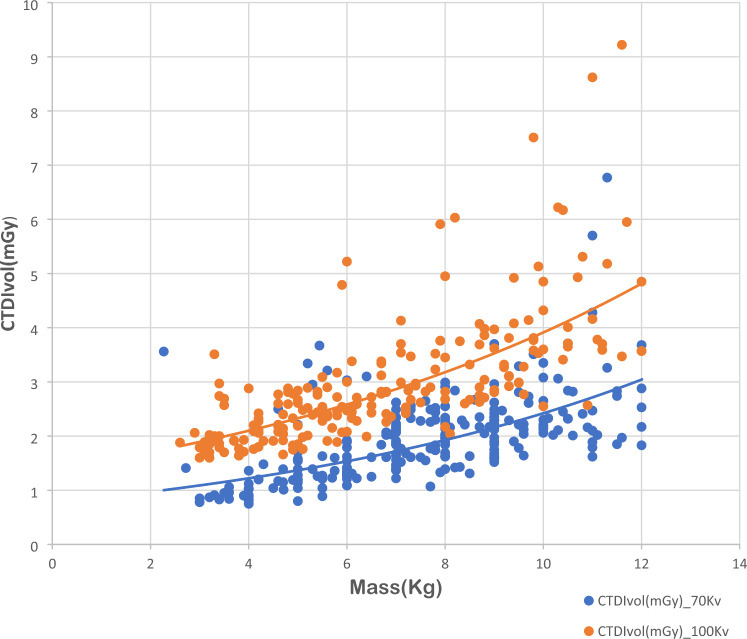
Computed tomography dose index (CTDI_vol_) values for paediatric cardiac computed tomography examinations as a function of patient mass for two different protocols based on variation of tube voltage (70 and 100 kVp)

**Fig. 2:**
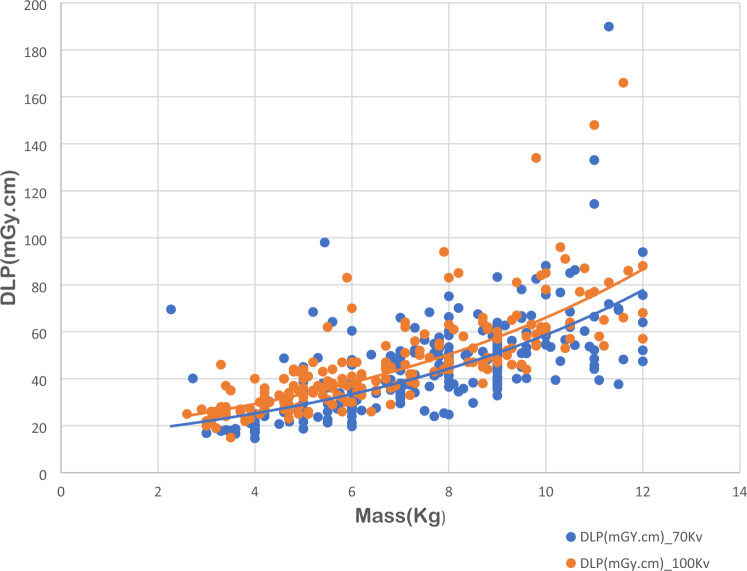
Dose length product (DLP) values for paediatric cardiac computed tomography examinations as a function of patient mass for two different imaging protocols based on variation of tube voltage (70 and 100 kVp)

It is known that during the infants’ cardiac computed tomography, the patients’ doses, in addition to their diagnostic image quality, are largely affected by potential voltage (kVp) and infant mass (kg). Figures 1 and 2 show the CTDI_vol_ and DLP values as a function of patient mass at tube voltages of 70 and 100 kVp, respectively.

The results confirm that CTDI_vol_ and DLP values increase with the patient mass. Interestingly, significant differences were found for different tube voltages, for CTDI_vol_ rather than DLP. Data shown in both figures demonstrate that CTDI_vol_ and DLP were correlated with patient body mass in an exponential manner, for different tube voltages (Trattner et al. 2018); (Järvinen et al. 2011, 2015); (Brady et al. 2012).

Table 1 shows median, mean, 3rd quarter (DRL), and dose reference range (DRR), for both CTDI_vol_ and DLP and both tube voltages (70 and 100 kVp). The mean of CTDI_vol_ and the standard deviation (SD) were 1.9 ± 0.7 mGy (range 0.7–6.7) and 2.9 ± 1.2 mGy (range 1.6–9.2) at 70 and 100 kVp tube voltage, respectively. The DLP mean and corresponding standard deviation (SD) were 45.2 ± 20.5 mGy.cm (range 14–189) and 45.9 ± 21.2 mGy.cm (range 15–166) at 70 and 100 kVp tube voltage, respectively. The mean ED based on DLP and the standard deviation (SD) were 1.17 ± 0.53 mSv (range 0.4–4.9) and 1.19 ± 0.55 mSv (range 0.4–4.3) at 70 and 100 kVp tube voltage, respectively.

**Table 1:** Dosimetric quantities of paediatric cardiac computed tomography examinations for two different protocols based on variation of tube voltage (70 and 100 kVp)

CT protocol	No. of patients	CTDI_vol_ (mGy)				DLP (mGy.cm)			
		DRL (75%)	Median	Mean ± SD (min–max)	DRR (IQR)	DRL (75%)	Median	Mean ± SD (min–max)	DRR (IQR)
Protocol 1 (70kVp)	245	2.3	1.9	1.9 ± 0.7 (0.7–6.7)	0.9 (1.4–2.3)	54.6	45	45.2 ± 20.5 (14–189)	24.1 (30.5 – 54.6)
Protocol 2 (100kVp)	208	3.5	2.7	2.9 ± 1.2 (1.6–9.2)	1.3 (2.2–3.5)	55	42	45.9 ± 21.2 (15–166)	23 (32–55)
Total	453	2.8	2.2	2.4 ± 1.1 (0.7–9.2)	1.1 (1.7 − 2.8)	54.8	43.5	45.6 ± 20.8 (14–189)	23.9 (30.9–54.8)

CT computed tomography, CTDI_vol_ volume computed tomography dose index, DLP dose–length product, DRL dose reference level, DRR diagnostic reference range [Q_3_ (75%) – Q_1_ (25%)], SD standard deviation

**Fig. 3:**
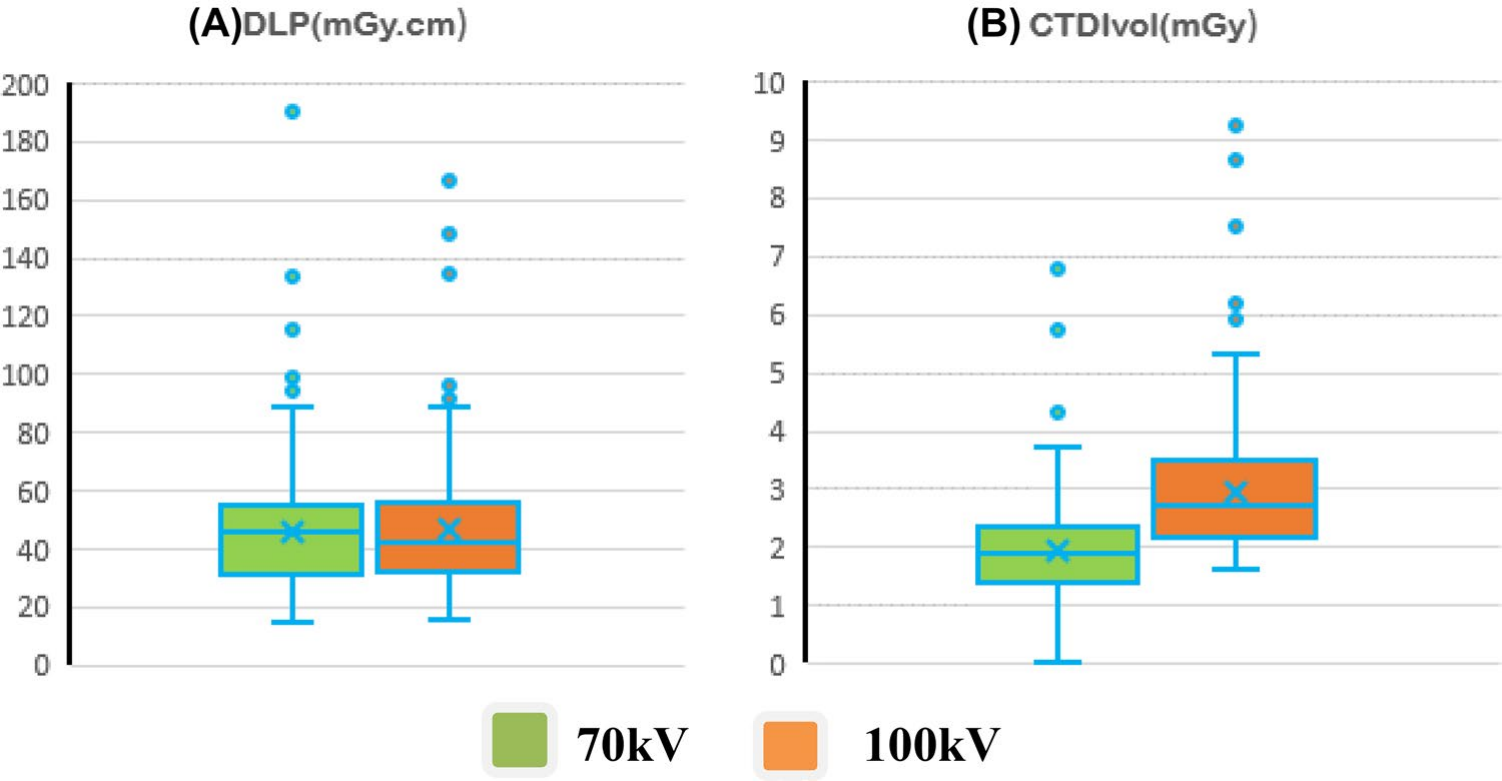
Box-and-Whisker plots of dosimetric quantities **A** dose length product (DLP) and **B** volume computed tomography index (CTDI_vol_) for two different protocols based on variation of tube voltage (70 and 100 kVp) of paediatric cardiac computed tomography examinations

Figure 3 shows box-and-whisker plots for CTDI_vol_ and DLP for tube voltages of 70 and 100 kVp, which were used to compare the impact of tube voltage on the proposed DRL based on clinical indications for cardiac paediatric patients. As can be seen in Table 1, DRLs for CTDI_vol_ were 2.3 and 3.5 mGy at 70 and 100 kVp tube voltages, respectively. The DRR [Q3(75%)–Q1(25%)] based on CTDI_vol_ were 0.9 (1.4–2.3) and 1.3 (2.2–3.5) mGy at 70 and 100 kVp tube voltage, respectively, while DRLs for DLP were 54.6 and 55 mGy at 70 and 100 kVp tube voltage, respectively. The DRR [Q3(75%)–Q1(25%)] based on DLP were 24.1 (30.5–54.6) and 23 (32–55) mGy.cm at 70 and 100 kVp tube voltage, respectively.

Because DRLs for CCT in children have yet to be established, this study aimed to establish provisional local DRLs for paediatric CCT. Consequently, a preliminary assessment was required. This resulted in the third quartile value of the dose distribution was 2.8 mGy and 54.8 mGy.cm in terms of CTDI_vol_ and DLP, respectively (Table 1). Similarly, the DRR [Q3(75%)–Q1(25%)] values were 1.1 (1.7–2.8) mGy and 23.9 (30.9–54.8) mGy.cm based on CTDI_vol_ and DLP, respectively.

## Discussion

A low tube voltage offers two advantages: a high contrast-to-noise ratio that gives iodinated cardio-vascular contrast, and a low contrast amount and radiation dose. In general, 70 kVp (if available) is enough for most infants below an age of 10 years (Vawda et al. 2015). As a result, the integration of CCT imaging protocols based on clinical indications with a proper tube voltage is critical for paediatric CHD, to maintain adequate diagnostic image quality while significantly reducing patient dose and, thus, cancer risk.

Since data on CCT CHD examinations of children is scarce, there is a lack of regional or international DRL standardization. Therefore, the establishment of DRLs especially for newborns and infants is a challenge, as well as the establishment of an efficient method to manage paediatric radiation doses and optimize CCT imaging protocols based on patient mass.

Data on CTDIvol and DLP as a function of patient mass are considered as efficient illustrations to present and compare DRLs for paediatric CCT imaging modalities (Rigsby et al. 2018).

Interestingly, the present study demonstrates the impact of CT imaging protocols at different tube voltage (70 and 100 kVp) on the feasibility of establishing local (institutional) DRLs for cardiac patients (newborns and infants) based on clinical indications. Therefore, various technical parameters in scanning protocols such as tube voltage, scan range, scan time, and tube current modulation, should be carefully chosen to design scanning protocols targeted to clinical indications, patient age and patient mass, to optimize scanner performance including maintaining sufficient diagnostic image quality while minimizing radiation exposure.

The DRLs for CCT examinations in terms of CTDI_vol_ (mGy) and DLP (mGy.cm) were 2.3 and 3.5, and 54.6 and 55, at tube voltages 70 and 100 kVp, respectively. The results obtained revealed that DRLs’ differences observed for different tube voltages were statistically significant in terms of CTDI_vol_ (Mann-Whiney test, *p* = 0.001), but statistically non-significant (Mann-Whiney test, *p* = 0.856) in terms of DLP. This is primarily because, in the present study, the investigated children showed different scan lengths: long scan ranges similar to those in chest CT (23.4 cm) and short scan ranges similar to those in coronary CT angiography (15.8 cm). Therefore, there is an urgent necessity to update local DRLs values for CHD paediatric examinations, based on CTDI_vol_ rather than DLP, to optimize the patient dose for radiation safety and the CCT imaging protocol to establish reasonable diagnostic information.

However, both CTDI_vol_ and DLP dose descriptors can be used to compare between different CCT protocols based on patient mass. The evaluation of effective dose (ED) in paediatric CCT is crucial for radiation protection purposes. Trattner et al. derived cardiac adult conversion factors to estimate ED from DLP in CCT (Trattner et al. 2018). Using adult conversion factors for calculating ED for paediatric patients can have limitations due to differences in size, anatomy, and physiology between adult and paediatric patients. In the current study, as well as in previous studies (Aamry et al. 2020; Ali et al. 2019; Ghoshhajra et al. 2014; Hustings et al. 2022), cardiac k factor based on ICRP recommendations and Trattner et al. (ICRP 007); (Trattner et al. 2018) was used, because the cardiac k conversion factor for paediatrics was unavailable.

In Table 2, patient EDs obtained in the current study are compared with those reported in the literature. The estimated EDs for CHD obtained in the present study for paediatric patients were lower compared with those reported in previous publications; this can be due to the lower tube potential used in the present study, and due to technological advances of the CCT imaging modality, especially the ATCM feature. The difference in ED values observed for 70 and 100 kVp was non-significant. This small difference could be attributed to the larger scan range at 70 kVp (23.4 cm) than at 100 kVp (15.8 cm). Therefore, CTDIvol is more effective than DLP to compare between different CT protocols.

**Table 2:** Comparison of effective dose obtained in the current study with results of pervious published studies

Author	No. of patients	No. of detectors (CT modality)	Tube voltage (kVp)	DLP (mGy.cm)	ED (mSv)	Conversion factor (mSv.mGy^−1^)
Our study	453	64/128	70–100	45.6 ± 20.8 (14 – 189)	1.2 ± 0.5 (0.4–4.9)	0.026
Aamry et al. (2020)	147	64/128	80–140	411.6 ± 225	11.5	0.028
Ali et al. (2019)	236	64/640	80–120	807	21	0.026
Hollingsworth et al. (2007)	Phantom	16	80–120	41.9–1344	7.4–28.4	0.021
Habib Geryes et al. (2016)	140	64	80–100	189.2 ± 215	5.3 ± 5	0.028
Westra et al. (2014)	10	16/64/128	80–120	185.7	2.6 ± 2.3 (0.4–7.9)	0.014
Ghoshhajra et al. (2014)	50	64/128	80–120	211.5 (64.0–648.0)	6.1 (2.5–10.6)	0.028
Hustings et al. (2022)	52	192/128	70	55.4 ± 18.8 (6.2–488.0)	1.3 ± 0.4 (0.2–10.9)	0.022
Nakada et al. (2016)	385	64	100	171 (63–1444)	6.7 (2.5–56.3)	0.014

DLP dose length product, ED effective dose, expressed as (mean ± standard deviation) (min–max)

*Conversion factor at 70 kVp was not available; therefore, the conversion factor at 80 kVp was used

Because there were different average scan ranges for examinations of the same region (cardiac), i.e., there was a short scan range for examination of heart structure and coronary arteries and a large scan range for examination of the entire chest, LDRL should ideally be determined for each individual protocol to allow for optimisation and tailoring to individual imaging needs.

Because in the present study only patients from one tertiary paediatric hospital were included, the findings obtained may not apply to all paediatric CT examinations across the country. Further studies are therefore crucial to develop a national DRL and update the current local DRL (Ploussi et al. 2020; Nakada et al. 2016).

## Conclusion

Recent studies have raised concerns about the implementation of DRLs for cardiac paediatric CT examinations. However, to the best of the authors’ knowledge no standardized local, regional, or international DRLs for paediatric CHD are available, especially for newborn and infants. Consequently, the DRLs for paediatric CCT are urgently required to allow for acceptable diagnostic image quality and dose reduction for radiation protection safety issue. The present study contributed toward the assessment of local LDRLs for paediatric CT cardiac imaging at a tertiary care children’s hospital. Specifically, the impact of different tube voltages on the proposed DRLs of paediatric CCT examinations was investigated. Assessing local practises is very important to understand the role of different factors and to improve medical staff awareness. The obtained results reveal that the local CTDI_vol_ and DLP values were 2.8 mGy and 54.8 mGy.cm, respectively, while the mean ED was 1.2 mSv. There was a statistically significant difference (Mann–Whitney, *p* = 0.001) between DRL values in terms of CTDI_vol_, for different tube voltages. Therefore, further research should be conducted to update the current DRLs and develop national DRL for paediatric cardiac CT imaging.

## Data Availability

The data is available upon the request of the journal.
